# A Disordered Region in the EvpP Protein from the Type VI Secretion System of *Edwardsiella tarda* is Essential for EvpC Binding

**DOI:** 10.1371/journal.pone.0110810

**Published:** 2014-11-17

**Authors:** Wentao Hu, Ganesh Anand, J. Sivaraman, Ka Yin Leung, Yu-Keung Mok

**Affiliations:** 1 Department of Biological Sciences, 14 Science Drive 4, National University of Singapore, Singapore, Singapore 117543; 2 Department of Biology, Faculty of Natural and Applied Sciences, Trinity Western University, Langley, British Columbia, Canada V2Y 1Y1; 3 State Key Laboratory of Bioreactor Engineering, East China University of Science and Technology, Shanghai, China 200237; The Chinese University of Hong Kong, China

## Abstract

The type VI secretion system (T6SS) of pathogenic bacteria plays important roles in both virulence and inter-bacterial competitions. The effectors of T6SS are presumed to be transported either by attaching to the tip protein or by interacting with HcpI (haemolysin corregulated protein 1). In *Edwardsiella tarda* PPD130/91, the T6SS secreted protein EvpP (*E. tarda* virulent protein P) is found to be essential for virulence and directly interacts with EvpC (Hcp-like), suggesting that it could be a potential effector. Using limited protease digestion, nuclear magnetic resonance heteronuclear Nuclear Overhauser Effects, and hydrogen-deuterium exchange mass spectrometry, we confirmed that the dimeric EvpP (40 kDa) contains a substantial proportion (40%) of disordered regions but still maintains an ordered and folded core domain. We show that an N-terminal, 10-kDa, protease-resistant fragment in EvpP connects to a shorter, 4-kDa protease-resistant fragment through a highly flexible region, which is followed by another disordered region at the C-terminus. Within this C-terminal disordered region, residues Pro143 to Ile168 are essential for its interaction with EvpC. Unlike the highly unfolded T3SS effector, which has a lower molecular weight and is maintained in an unfolded conformation with a dedicated chaperone, the T6SS effector seems to be relatively larger, folded but partially disordered and uses HcpI as a chaperone.

## Introduction

The type VI secretion system (T6SS) was first discovered as an essential bacterial secretion system for bacterial pathogenesis [1], [2], and its activity is tightly controlled by both environmental and bacterial regulatory factors [3]. Accumulated evidence now shows that the T6SS of pathogenic bacteria have dual roles in both pathogenicity and inter-bacterial competition, and that the T6SS is an effective weaponry against other Gram-negative bacteria in the living habitat [4]. As the prey will usually fight back by secreting toxins from its own T6SS, this process of inter-bacterial competition is termed as “T6SS dueling” [5].

The T6SS core apparatus assembles from 13 “core components” proteins, from TssA-M (type six sub-units), in addition to Hcp (haemolysin corregulated protein), VgrG (valine-glycine repeat protein G), the ClpV AAA^+^ ATPase and the T4SS IcmF- and IcmH-like proteins [6]–[8]. These proteins are arranged in the form of two sub-assemblies: a dynamic bacteriophage-like structure and a cell-envelope-spanning membrane-associated assembly [9]. The phage-like complex is functionally related to the phage tail and comprises the Hcp (haemolysin corregulated protein) tube, the trimeric VgrG (valine-glycine repeat protein G) tip protein with a PAAR (proline-alanine-alanine-arginine) sharpened tip [10], and the sheath-containing TssB and TssC proteins [11], [12]. Extension and contraction of the sheath will drive the Hcp tube and the VgrG tip toward the host cell/bacteria to export T6SS effectors [13]. The trans-envelope complex contains a large base-plate with inner membrane components, TssL and TssM, and the outer membrane lipoprotein, TssJ, for anchoring the phage-like structure to the membrane [14]–[16]. TssK is a trimer that connects the two systems by directly interacting with TssL, HcpI and TssC [17].

T6SS has two currently known routes for effector proteins delivery. The first route is through the “evolved VgrG proteins”, where the VgrG tip protein harbors an additional effector domain fused to the C-terminus of the gp-5-like β-helix needle [18]. Such additional effector domains include an actin cross-linking domain, which fuses to VgrG [19]. Alternatively, the RhsA effector nuclease, a PAAR protein that recognizes the VgrG trimer and decorates the tip of the T6SS injectisome for export [20]. The second route is through the delivery of “classic toxins” into the host cell by passing through the HcpI channel [4], [21]. Recent findings, however, showed an alternate mechanism in which effector form a complex with the HcpI ring and injected as such. The hexameric HcpI ring is proposed to act as a chaperone and receptor for these and other effectors molecules [22]. In general, this second class of effector binds to residues on the inside of the HcpI hexamers [21].

*Edwardsiella tarda* is a recognized fish pathogen that has been shown to also cause gastrointestinal infections in human [23]. The EvpP (*E. tarda* virulent protein P) protein is essential for *E. tarda* virulence and has been identified as one of the three secreted proteins—together with EvpC (hexameric and HcpI-like) [24] and EvpI (VgrG-like)—from the T6SS of *E. tarda*. The secretion of EvpC and EvpI is mutually dependent, and both are required for the secretion of EvpP, with evidence to show binding between EvpP and EvpC [25]. In the T6SS gene cluster, EvpP is located at the first ORF and its expression is tightly regulated by Fur and the PhoB-PhoR two-component system, which sense iron and phosphate, respectively [26]. The promoter of *evpP* binds directly to the transcription activator EsrC, which in turn is inhibited by the direct interaction between EsrC and Fur protein. EvpP has orthologs in only two other genomes (*E. italuri* and *Vibrio* MED222 [27]) and has no known function or sequence homology with other existing proteins. EvpP is not an anti-bacterial peptidoglycan peptidase like many other known T6SS effectors and could represent a potential novel class of T6SS effector.

Using limited protease digestion, nuclear magnetic resonance (NMR) experiments and amide proton exchange mass spectrometry, we found that EvpP is highly disordered with the disordered region mapping to the middle of the protein and shown to link two relatively folded regions. The highly disordered nature of the protein and the known interaction between EvpP and EvpC suggests that EvpP is a T6SS secreted substrate which translocates through interaction with EvpC. Further experiments are needed to confirm whether EvpP is a *bone fide* T6SS effector.

## Materials and Methods

### Protein expression and purification

Wild type and mutant *evpP* sequences were cloned between BamH I and EcoR I sites of the pET-M vector (derived from pET32-a, Novagen; Madison, WI) and then transformed into *E. coli* BL21 (DE3) competent cells. The cells were allowed to grow in LB with ampicillin at 37°C until the O.D. reached 0.6. IPTG (0.4 mM) was added into the media to induce protein expression and the cells were grown overnight at 20°C. The cells were then harvested in 20 mM Tris buffer, pH 7.0, sonicated and centrifuged. The supernatant was collected and passed through a Ni-NTA affinity column. Expression of ^15^N- or ^13^C-labeled protein was carried out in similar conditions as mentioned above, except that the cells were grown in M9 minimal medium supplemented with ^15^N-labeled ammonium chloride and/or ^13^C-labeled D-glucose. To obtain a single labeled sample (either ^15^N-labeled or ^13^C-labeled sample), cells were grown in 1×M9 minimal medium in the presence of ^15^N-ammonium chloride (Cambridge Isotope Laboratories; Andover, MA) or with ^13^C-glucose (Cambridge Isotope Laboratories) as the sole nitrogen or carbon source. For double labeled ^15^N- and ^13^C-sample, 1×M9 minimal medium with ^15^N-ammonium chloride and ^13^C-glucose (Cambridge Isotope Laboratories) was used as the sole nitrogen and carbon sources, respectively.

### Dynamic light scattering

Dynamic light scattering was carried out for both EvpP and its mutant EvpP-P143T using DynaPro Dynamic Light Scattering (Protein Solutions LLC; Joplin, MO) machine linked to a Temperature Controlled MicroSampler (Protein Solutions). Twenty microliters of protein sample (50 µM) was loaded into a quartz cuvette with a path length of 15 mm (Protein Solutions). Data were recorded and analyzed using the Instrument Control Software for Molecular Research (Protein Solutions).

### Limited protease digestion

Limited protease digestion (enzymes used were trypsin, chymotrypsin, elastase, thermolysin) was carried out for both EvpP and its mutant EvpP-P143T using protein samples in their native buffer (20 mM Tris-Cl, pH 7) with different enzymes at different ratios (usually 1∶50, 1∶100 or 1∶200). Both the protein sample and the enzyme stock were prepared as 1 mg/ml. Ten microliters of enzyme solution was added into 1 ml of protein sample to form the reaction mixture. Aliquots were removed from the reaction mixture every 10 or 15 min and mixed with an equal volume of 2×SDS sample buffer and boiled at 100°C for 5 min. All of the aliquots were analyzed on SDS-PAGE.

### Mass spectrometry and N-terminal sequencing

Trypsin-digested EvpP samples were blotted onto a MALDI target plate with sinapinic acid as a matrix. The molecular weight of each component in the complex was determined using Voyager-DE STR Mass Spectrometer (GE Healthcare; Buckinghamshire, UK). A voltage of 2100 V and laser intensity of 2500 was used for the experiment. For N-terminal sequencing, protein samples were separated on a 15% SDS-PAGE or 15% Tricine-SDS-PAGE gels, and then blotted onto PVDF membranes (EMD Millipore, Billerica, MA) using a Mini Trans-blot Electrophoretic Transfer Cell (Bio-Rad; Hercules, CA) at 4°C at a voltage of 90 V for 90 min or until complete transfer of protein bands onto the membrane. Protein bands on the membrane were visualized by coomassie blue staining and excess stain on the membrane was removed by washing with a 50% methanol solution. Membrane-bound protein bands were excised and N-terminal residues were fragmented using Procise Protein sequencing system (Applied Biosystems, Life Technologies; Carlsbad, CA). Data were collected and analyzed using SequencePro Data Analysis Application software v2.1 (Applied Biosystems, Life Technologies).

### Nuclear magnetic resonance and backbone assignment

3D and 4D heteronuclear NMR experiments were carried out to assign the backbone chemical shifts for EvpP-P143T. All NMR experiments were carried out on a Bruker AVANCE 800 MHz spectrometer (Madison, WI) equipped with a cryoprobe. Samples were loaded into a 5-mm NMR tube and all experiments were carried out at 297 K. All data acquired were processed using NMRPipe [28] and analyzed using NMRDraw [28], SPARKY [29] and NMRspy (Zheng, Yu; Yang, Daiwen. NMRspy, National University of Singapore).

### HDX-MS (hydrogen-deuterium exchange mass spectrometry)

Sample protein was exposed to 100% D_2_O buffer for various incubation times and the reaction quenched by trifluoroacetic acid prior to mass spectrometry. In order to increase the resolution in terms of amino acid sequence, the quenched sample was subjected to pepsin digestion. Prior to ESI mass spectrometry, the peptides were seperated on an HPLC column to further increase resolution. The whole process was carried out in Waters nanoACQUITY UPLC Hydrogen Deuterium Exchange (HDS) System (Waters Corporation; Milford, MA). Data were recorded and analyzed by ProteinLynx software (ProteinLynx Global Server, Waters Corporation). The peptide spectra assignment for each identified peptide in the undeuterated sample as well as in the subsequent deuterated samples was carried out using the DynamX software (Waters Corporation). The output file was converted to a Microsoft Excel format (Microsoft, Redmond, WA) for further data analysis. The back-exchange constant used for calculating the exchange rate was 1.49.

### Glutathione-S-transferase (GST) pull-down assay

The buffer for all EvpP and its truncation mutants was exchanged to PBS before GST pull down assay. Glutathione-sepharose 4B beads (50 µl) (Amersham Biosciences, GE Healthcare, Piscataway, NJ) was added into an Eppendorf tube (Eppendorf, Hamburg, Germany) and was washed at least three times with 1 ml of 1×PBS. GST-EvpC (100 µg) or GST protein alone as a control was added into the tube and the mixture was topped up to 500 µl with 1×PBS. The protein∶bead mixture was incubated at 4°C for at least 1 h. The beads were washed with 1 ml of 1×PBS three times. EvpP or its truncation mutants (200 µg) was added into the tube and the reaction volume was topped up to 500 µl by 1×PBS. The mixture was incubated at 4°C for at least 2 h with mixing. Finally, the beads were washed with 1 ml of 1×PBS to remove unbound or loosely bound proteins, and the beads suspended in 100 µl of 2×SDS sample buffer. The suspension was boiled for 5 min, centrifuged at 13,000 rpm for 10 min, and 20 µl of the supernatant was analyzed on SDS-PAGE.

## Results

### EvpP purified as a dimeric protein

**Figure 1:**
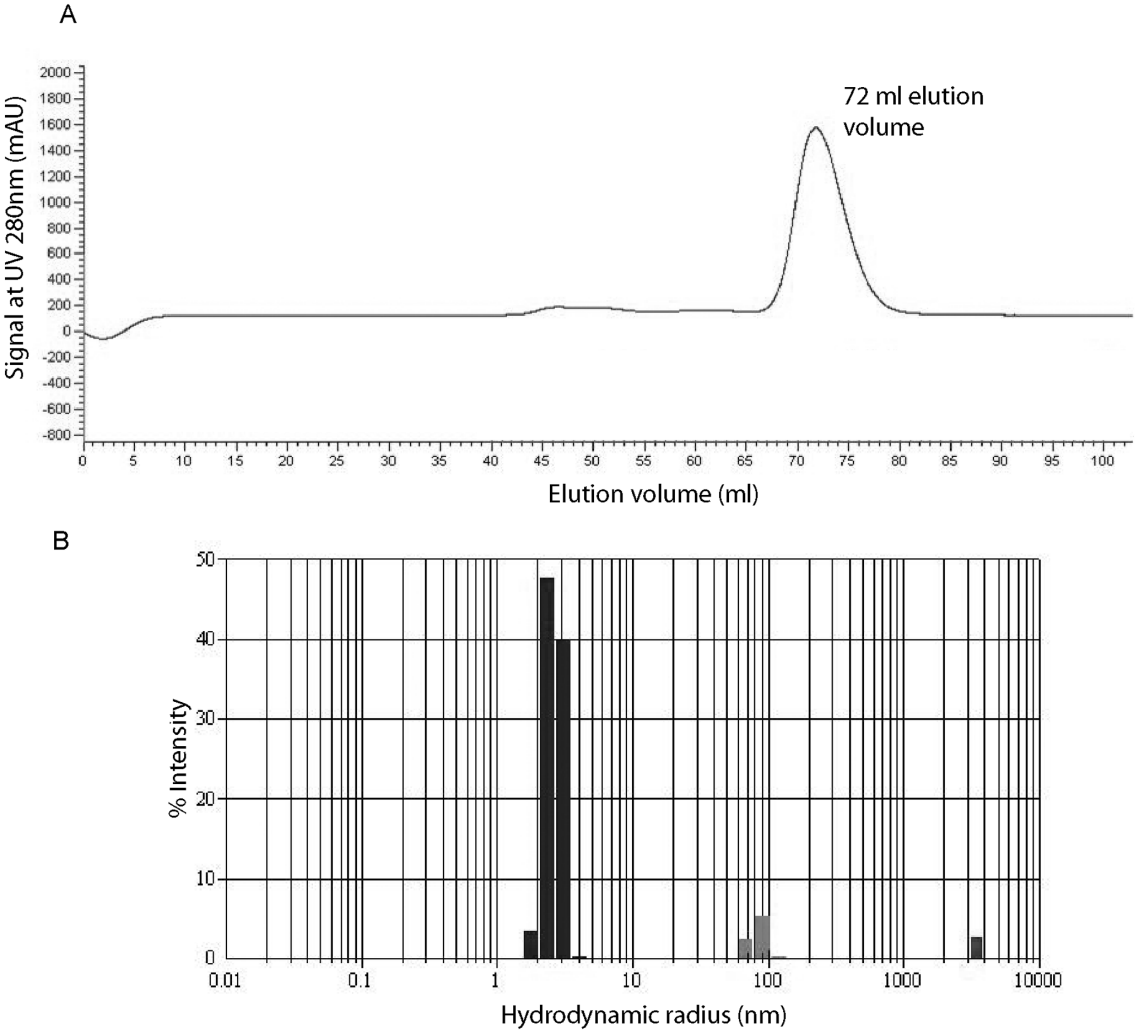
EvpP is a dimeric protein in solution. (A) The recombinant wild type EvpP purified as a dimeric protein with an elution volume of 72 ml from the Superdex 75 gel filtration column (HiLoad 16/60, GE Healthcare). (B) Dynamic light scattering data showing that the hydrodynamic radius of EvpP ranged from 2.92 nm to 3.09 nm, with a mean value of 3.01 nm. The estimated molecular weight ranged from 41.2 kDa to 47.3 kDa, with mean value of 44.4 kDa (data not shown).

EvpP from *E. tarda* strain PPD130/91 is a 185-amino acid protein with a molecular weight of 20.3 kDa and a theoretical isoelectric point of 9.47. 6× His-tagged EvpP was over-expressed in *E. coli* BL21 (DE3) as a soluble protein and purified through Ni-NTA affinity column. The purified EvpP protein eluted from the Superdex 75 gel filtration column (HiLoad 16/60, GE Healthcare) at a volume of ∼72 ml. Molecular weight standards ovalbumin (44 kDa) and myoglobulin (17 kDa) on the same column eluted at 63 ml and 83 ml, respectively. This elution comparison suggests that EvpP could be a dimer and the slight increased elution volume may be due to the flexible conformation of the protein (Figure 1A). To confirm the dimeric nature of EvpP, we performed dynamic light scattering (DLS) experiments to study the oligomeric state of proteins in solution. At 20°C, EvpP showed a profile corresponding to a dimeric molecule. The hydro-dynamic radius of EvpP was determined to be from 2.92 nm to 3.09 nm (mean, 3.01 nm; n = 11 readings) and the estimated corresponding molecular weight ranged from 41.2 kDa to 47.3 kDa (mean, 44.4 kDa; n = 11 readings); this measured molecular weight is twice that of the theoretical molecular weight for 6× His-tagged EvpP (22.0 kDa) (Figure 1B). For most of the readings, the polydispersity index (PDI) lies within the range from 0.0 to 0.1 (% polydispersity from 0% to 31%), suggesting that EvpP is a mono-dispersed dimeric species under the conditions used (Figure S1 in File S1). In addition, cross-linking experiments using glutaraldehyde were performed showing that the dimeric form of EvpP is the main cross-linked species (Figure S2 in File S1).

### EvpP limited protease digestion generated a folded core comprising two fragments

**Figure 2:**
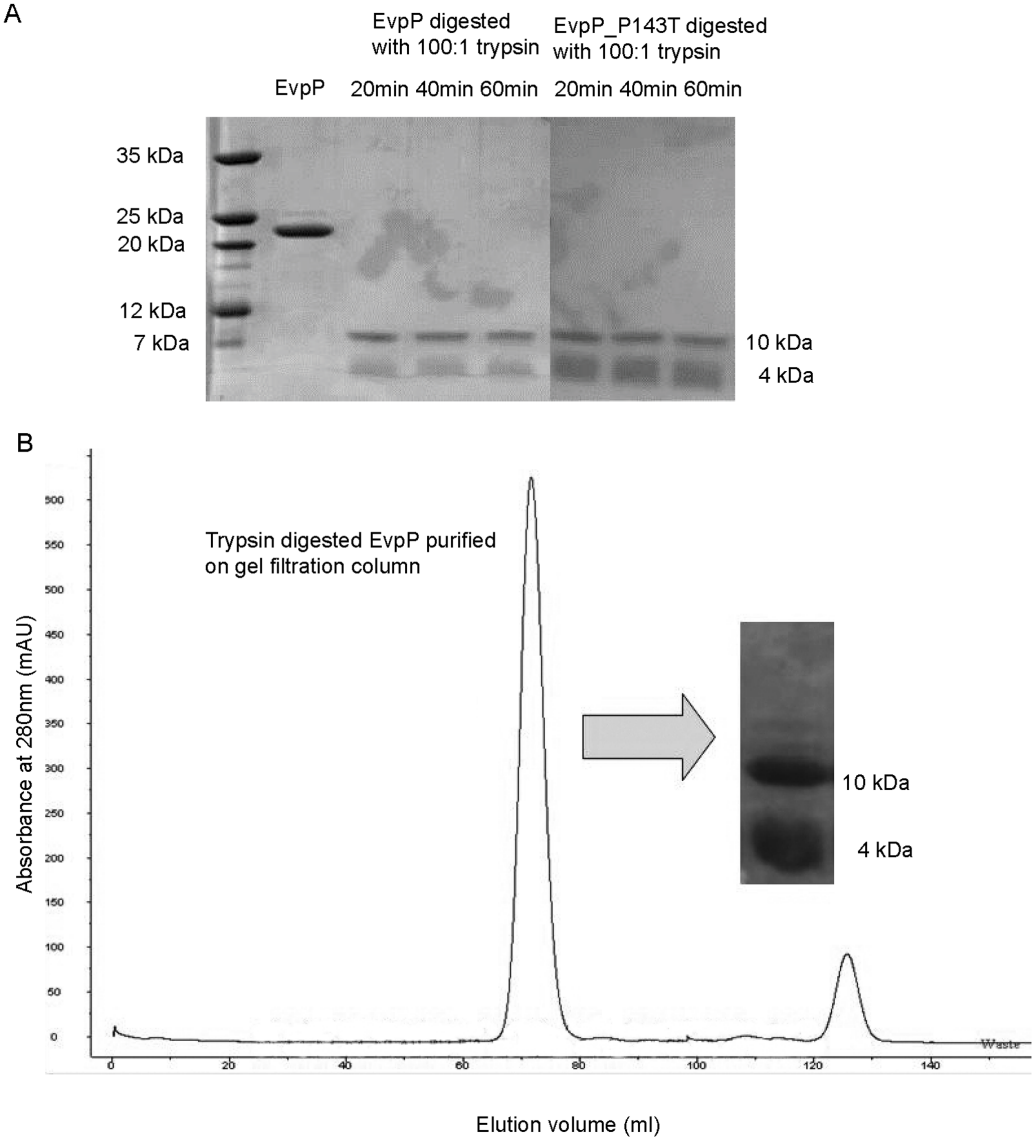
Limited trypsin digestion of EvpP and EvpP P143T. (A) Limited trypsin digestion of both wild type and P143T EvpP resulted in two protease-resistant fragments of 10 kDa and 4 kDa consistently within a digestion period of 20–60 min. (B) Gel filtration elution profile showed that the 10-kDa and 4-kDa fragments remain associated with each other after protease digestion.

**Figure 3:**
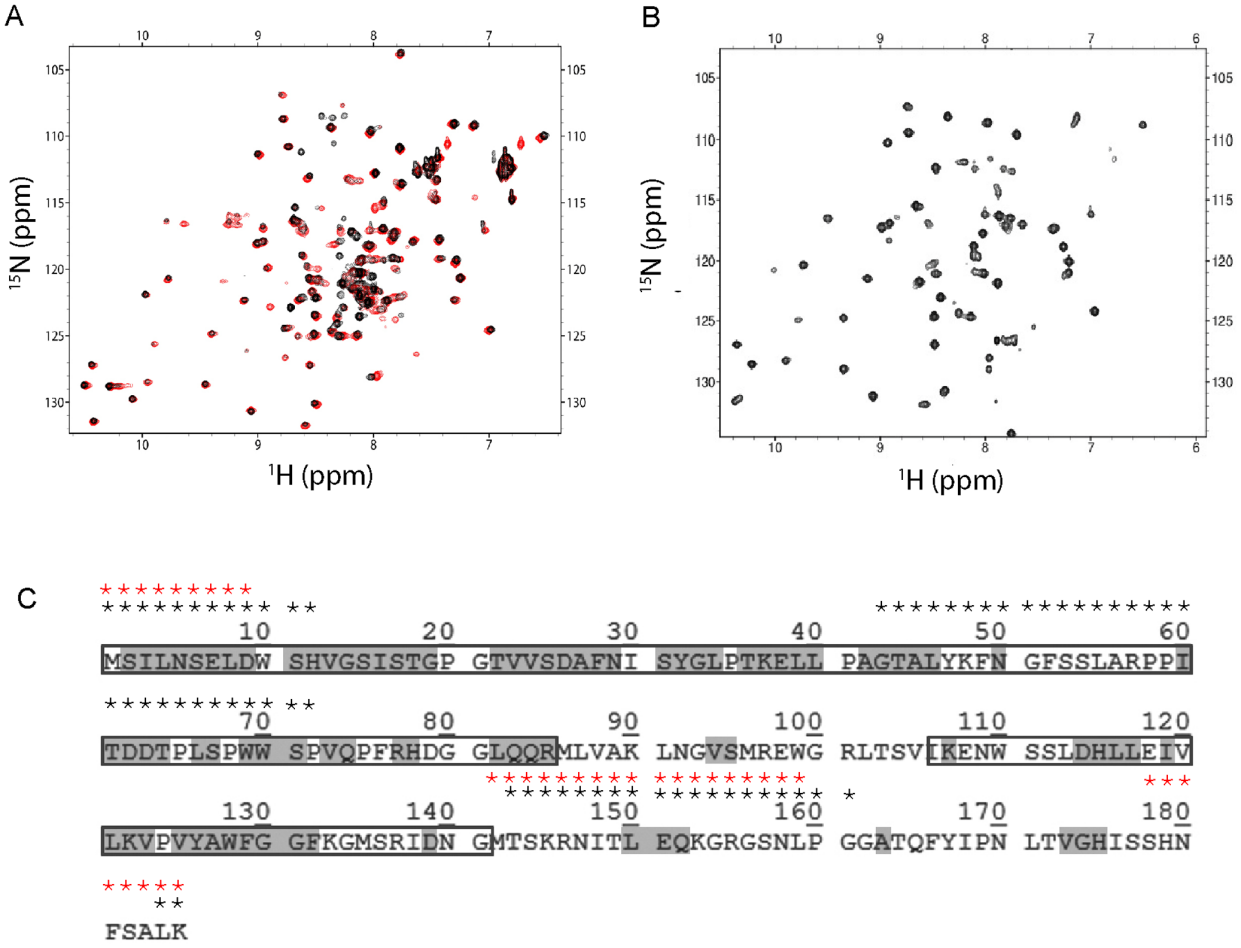
^1^H-^15^N HSQC spectra of wild type, P143T and trypsin digested EvpP. (A) Overlay of the ^1^H-^15^N HSQC spectra of wild type EvpP (black) and P143T EvpP (red). (B) ^1^H-^15^N HSQC spectrum of trypsin digested EvpP. (C) Sequence of P143T EvpP from *E. tarda* PPD130/91, with the boundaries of the 10-kDa (residues Met1 to Arg85) and 4-kDa (residues Ile106 to Gly141) protease-resistant fragments boxed. Residues that can be assigned by NMR experiments are shaded in grey. Residues that are predicted to be disordered by the software RONN [33] and PrDOS [34] are marked by black and red asterisks, respectively, above the sequence.

To ascertain if EvpP contained any disordered regions and to further map these regions, we performed limited protease digestion on EvpP using trypsin. At a protein∶trypsin ratio of 100∶1, trypsin reproducibly digested EvpP to two fragments of 10 kDa and 4 kDa as observed on SDS PAGE (Figure 2A). This digestion was completed within 20 to 30 min and further digestion was not observed even after 60 min. When analysed on gel filtration columns (HiLoad 16/60 Superdex 75), the two fragments were found to associate with each other and both eluted at a volume similar to that of native EvpP (Figure 2B). This suggested that the trypsin digestion retained EvpP as a dimer (∼28 kDa) and that each monomer (∼14 kDa) contained folded regions from two different parts of the protein. Freshly digested EvpP also showed a ^1^H-^15^N HSQC NMR spectrum typical of a folded protein (Figure 3B) and the line widths of the cross-peaks are comparable to that of native EvpP, although the protein tended to denature after approximately 3 days (data not shown).

We next determined the boundaries of the 10-kDa and 4-kDa protease-resistant regions of EvpP by performing N-terminal sequencing on both fragments. The 10-kDa fragment N-terminal sequence was “GSELMSILN”, where “GSEL” is from the vector and “MSILN” corresponds to the first five residues of EvpP; this indicates that the N-terminal region of EvpP is resistant to tryptic digestion. Using electrospray mass spectrometry, we determined the molecular weight of this 10-kDa fragment as 9,614 Da. Combining this molecular weight and N-terminal sequencing data, the 10-kDa trypsin-resistant fragment was able to be mapped to residues Met1 to Arg85 of EvpP (Figure 3C). For the shorter 4-kDa fragment, our attempts at N-terminal sequencing were not successful but the molecular weight of the fragment was determined as 4,121 Da. Instead of N-terminal sequencing, we performed peptide identification on the 4-kDa fragment using MALDI-TOF-TOF tandem mass spectrometry with trypsin digestion. Two peptides were chosen and their molecular weights (1,796 Da and 1,270.7 Da) were found to match the EvpP sequences “ENWSSLDHLLEIVLK” (residues Glu108 to Lys122) and “VPVYAWFGGFK” (residues Val123 to Lys133), respectively. Based on the determined molecular weight of the fragment, we deduced the boundaries of this 4-kDa fragment to be from residues Ile106 to Gly141 of EvpP (Figure 3C).

### NMR backbone assignment of P143T EvpP

The wild type EvpP contained too many disordered regions to be crystallized for structure determination. In addition, the NMR sample of EvpP at room temperature tended to precipitate steadily. Thus, we sought to obtain a more stable mutant of EvpP to carry out NMR experiments. Sequence alignment of EvpP from *E. tarda* PPD130/91 [2] with homologues from *E. tarda* EIB202, *E. tarda* 080813, *Aeromonas hydrophila* [30] and *E. tarda* FL6–60 [31] showed that residues Pro20, Leu34, Pro58, Leu91, Pro143 and Gly156 are unique to *E. tarda* PPD130/91. The corresponding residues in other homologues are Ala22, Gln36, Ser60, Gln93, Thr145 and Ser158, respectively (Figure S3 in File S1). The contrasting branching and flexibility properties of these residues would likely contribute to the differences in stability between EvpP from *E. tarda* PPD130/91 and that of other strains. Therefore, we performed site-directed mutagenesis at these locations (P20A, L34Q, P58S, L91Q, P143T and G158S) and found that one of the mutants, P143T, had a higher stability than the wild type EvpP, as determined with urea denaturation experiments (Figure S4 in File S1). The EvpP P143T mutant showed a similar digestion pattern as wild type EvpP in limited protease digestion using trypsin, suggesting that the same disordered regions still exist in the mutated protein (Figure 2A). The ^1^H-^15^N HSQC NMR spectrum of EvpP P143T was also similar to that of wild type EvpP (Figure 3A); however, the mutant was stable at room temperature, which thus allowed us to carry out sequential backbone assignment through various 3D and 4D NMR experiments.

**Figure 4:**
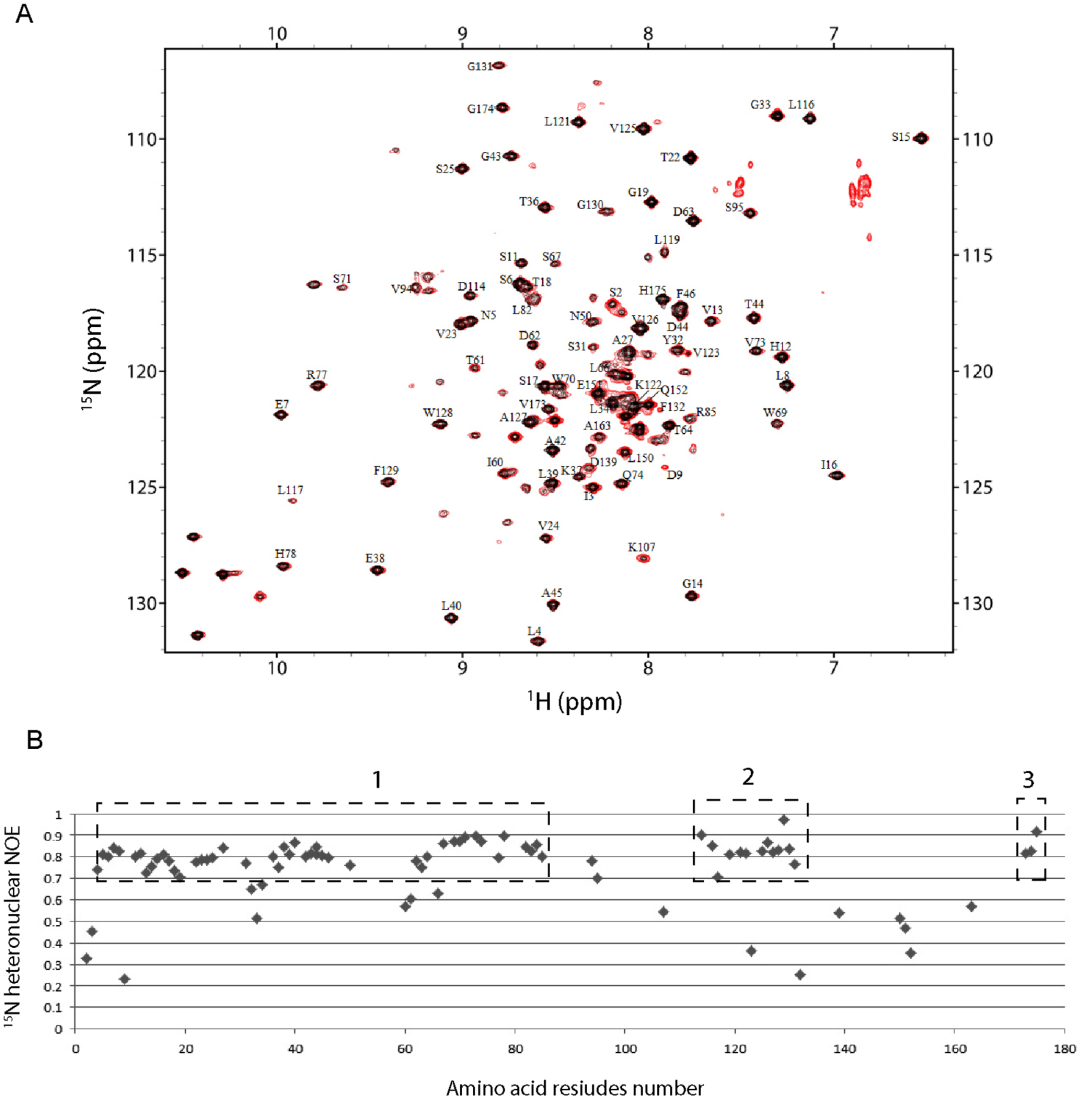
Heteronuclear NOE experiment on P143T EvpP. (A) Overlay spectra from heteronuclear NOE experiments showing cross-peaks from an experiment without proton saturation (red) and an experiment with proton saturation (black). The label next to each peak corresponds to sequential assignment of the residue. (B) A plot showing the relative ratio of peak intensities with proton saturation against those without proton saturation for all residues that can be assigned. Residue with a low value for the ratio is located at a disordered region. Boundaries of the three identified ordered regions are boxed by dashed lines and labeled Box 1, 2 and 3.

The theoretical number of backbone amide cross-peaks in the ^1^H-^15^N HSQC spectrum of His-tagged EvpP should be 190 peaks; however, only ∼50% of these peaks were able to be observed in the ^1^H-^15^N HSQC spectra of wild type EvpP (90 peaks) and P143T EvpP (96 peaks) (Figure 3A). This large number of missing peaks suggested that EvpP contains an extensive number of disordered regions, which undergo medium time scale conformational exchange. We attempted to assign P143T EvpP using standard through-bond triple resonance experiments; however, this was not successful because of the exceptionally short relaxation time of the protein. Subsequently, we adopted an NMR strategy that utilized both through-bond and through-space NMR experiments for resonance assignment. A set of five experiments were performed: ^15^N-edited HSQC, ^15^N-^13^C-edited HNCA, HN(CO)CA, 4D-NOESY and ^13^C-edited CCH-TOCSY. This strategy was previously designed for backbone and side chain assignment of large proteins without deuteration [32]. Using this method, we are able to assign 89 amide proton cross-peaks out of 96 detected cross-peaks from the ^1^H-^15^N HSQC of P143T EvpP (Figure 4A). The assigned residues were located mainly at the more-ordered and protease-resistant 10-kDa and 4-kDa regions (Figure 3C). This agrees with the notion that regions lacking HSQC peaks are likely to be flexible and more susceptible to protease digestion. One short region from residues Gly51 to Pro59 could not be assigned, suggesting that this region may also be undergoing medium time scale conformational exchange even though it is located inside the 10-kDa, protease-resistant fragment. Prediction of disordered regions in EvpP using the software RONN [33] and PrDOS [34] also picked up this unassigned region and another unassigned region from residues Met142 to Gly161 between the more ordered 10-kDa and 4-kDa regions (Figure 3C).

### Heteronuclear nuclear Overhauser effects (NOE) experiments verify ordered regions on P143T EvpP

To further verify whether the cross-peaks shown on the ^1^H-^15^N HSQC spectrum account for residues in the ordered regions, we performed heteronuclear NOE experiments using ^15^N-labeled P143T EvpP. The two HSQC-like spectra, with or without proton saturation, were superimposed for peak intensity comparisons (Figure 4A). A ratio of the peak intensity from the spectrum after proton saturation over that before proton saturation was obtained for each assigned residue. A ratio close to 1.0 represents a residue with an ordered backbone conformation located in a more rigid region. Residues with intensity ratios of ∼0.7–0.9 (as indicated in boxed regions 1, 2 and 3; Figure 4B) represent residues within more ordered regions. Residues within regions 1 and 2 are located within the 10-kDa and 4-kDa fragments, respectively, and are thus resistant to limited protease digestion. Residues in region 3 may represent a very short ordered region that cannot be resolved on SDS-PAGE, which is also resistant to limited protease digestion. Indeed, we observed two long-range NOEs from residues Val173 and Gly174 to residues Asp62 and Thr64 at the N-terminal ordered region (data not shown). Most of the assigned residues located outside of these three boxed regions have a peak intensity ratio lower than 0.6, which is in agreement with our supposition that these regions have a disordered conformation and are thus more susceptible to protease digestion (Figure 4B).

### HDX-MS experiment to map disordered regions on EvpP

**Figure 5:**
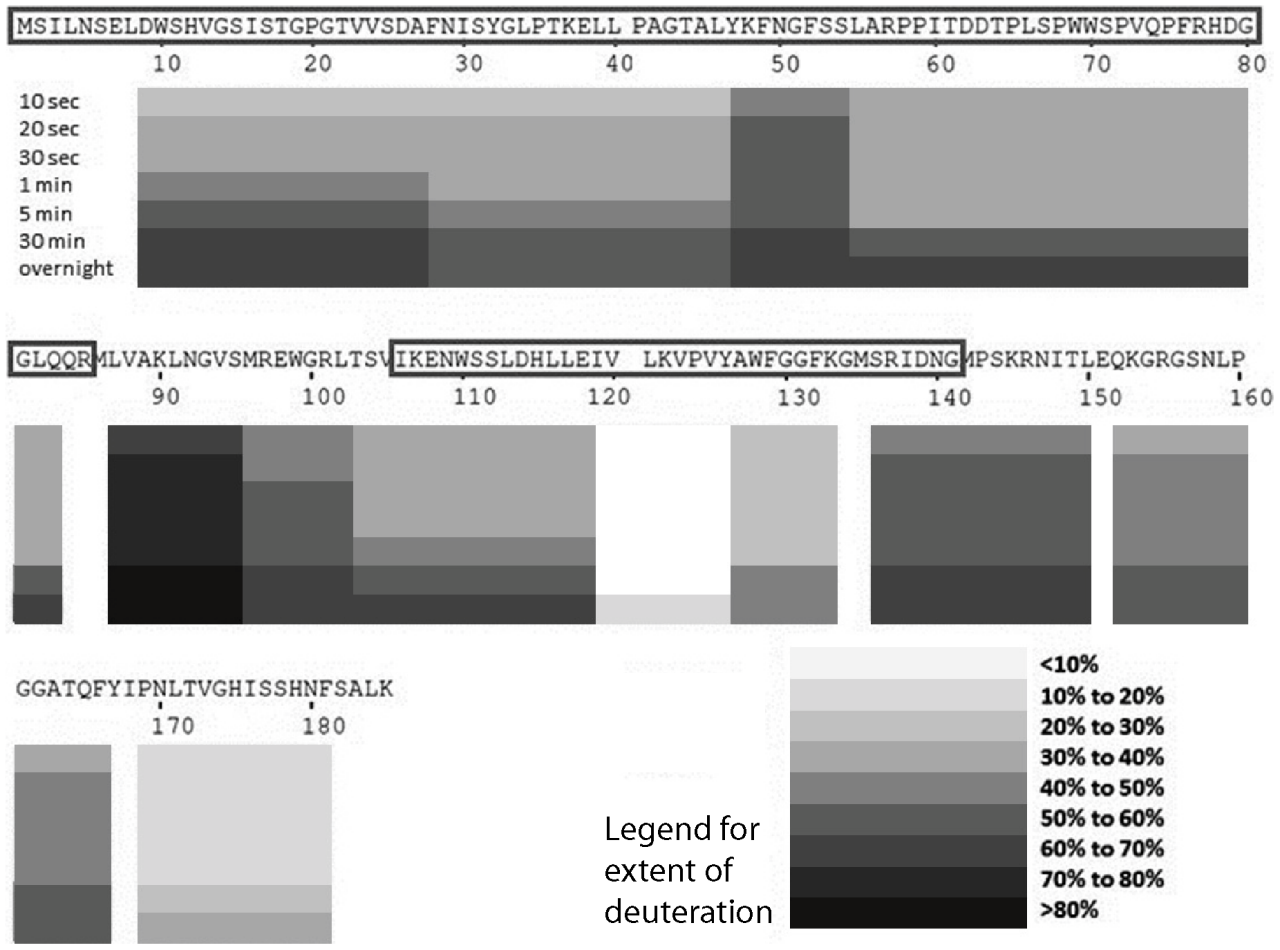
Hydrogen-deuterium (H/D) exchange heat map of wild type EvpP. The peptides from 12 subsets with the highest signal intensities were chosen from respective mass spectra. The extent of deuteration for the peptides exchanged for various time periods (from 10 sec to overnight) is plotted along the amino acid sequence of EvpP. The boundaries of the 10-kDa and 4-kDa protease-resistant fragments are boxed in the sequence of EvpP above the heat map.

Hydrogen-deuterium exchange mass spectrometry (HDX-MS) experiments [35]–[37] provide an alternative approach with which to probe the disordered regions in EvpP. The wild type EvpP generated a library of 117 peptide fragments with sequence coverage of 99.5%. In order to better depict the H/D exchange patterns of EvpP, a subset of 12 peptides with the highest mass spectrometry signal intensities was chosen from the peptide library to plot a “heat map” spanning the entire protein sequence with the least overlapping residues. The extent of deuteration for each peptide was plotted in a time-dependent manner, and the peptide was mapped against the sequence of the protein to help visualize the global pattern (Figure 5). The extensively deuterated peptides all pointed to three regions of EvpP that could be disordered. The most disordered of these stretched from residues Leu87 to Leu102. This region connects the 10-kDa and 4-kDa fragments and is susceptible to limited protease digestion. Extensive hydrogen/deuterium exchange was found among the two peptides in this region, with one of them showing as much as 80% deuteration. A second flexible region was observed from residues Arg136 to Phe166, which represents the region C-terminal to the 4-kDa fragment obtained from protease digestion. This region is also susceptible to limited protease digestion and has missing cross-peaks in the HSQC spectrum. The third flexible region ran from residues Lys48 to Ser54. This short stretch is located within the 10-kDa fragment obtained from protease digestion of EvpP. Residues from this region have missing cross-peaks from the HSQC spectrum and were unable to be assigned, suggesting conformational flexibility.

The heat maps also pointed to three regions that are more protected from deuteration. Firstly, a protected and presumably more-ordered region was found from residues Thr103 to Lys133, a region which overlaps with the 4-kDa fragment. The region spanning residues Val120 to Tyr126 represents the most ordered/buried region throughout the entire EvpP sequence, located at the middle of the 4-kDa fragment. A second region, corresponding to the 10-kDa fragment, was found in the N-terminal half of EvpP, running from residues Asp9 to Gln83. With the exception of residues Lys48 to Ser54, this region has a lower overall extent of hydrogen deuterium exchange as compared with the disordered regions, particularly during the initial time period of 10–30 sec. The last protected region was found from residues Pro169 to Phe181 at the C-terminus of EvpP. The extent of deuteration in this region was even less than most of the N-terminal regions. The presence of this protected region agrees with our findings from the heteronuclear NOE experiment that residues Val173 to His175 are ordered (Figure 4B, boxed region 3). Residues within this region could be interacting with residues from the N-terminal ordered region of EvpP through tertiary folding and provide stability.

### Interaction of truncation mutants of EvpP with GST-EvpC

**Figure 6:**
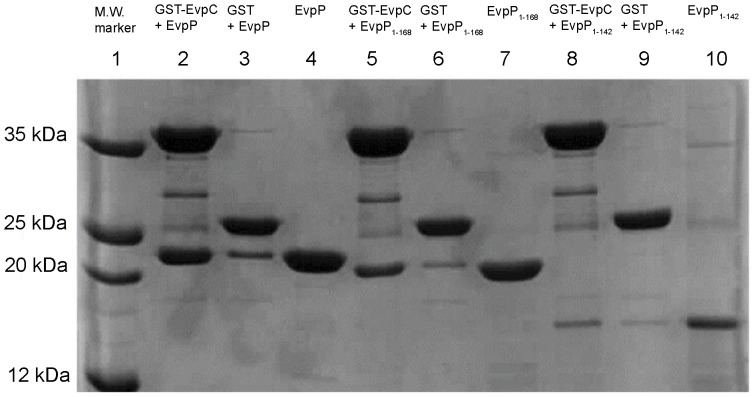
GST-EvpC pull-down assay for truncation mutants of EvpP. SDS-PAGE showing pull-down results using GST-EvpC and truncation mutants of EvpP. Lane 1: M.W. marker; lane2: GST-EvpC+EvpP; lane 3: GST+EvpP; lane 4: EvpP; lane 5: GST-EvpC+EvpP_1–168_; lane 6: GST+EvpP_1–168_; lane 7: EvpP_1–168_; lane 8: GST-EvpC+EvpP_1–142_; lane 9: GST+EvpP_1–142_; and lane 10: EvpP_1–142_. The region from residues Pro143 to Ile168 is essential for the interaction between EvpC and EvpP.

To determine if the disordered region is important for the binding of EvpP to EvpC, we generated C-terminal truncation mutants of EvpP and determined its binding to GST-EvpC (Figure 6). The GST-EvpC is neither in dimeric nor hexameric form and likely assumed an aggregated form. Removal of the C-terminal ordered region of EvpP (EvpP_1–168_) did not affect its binding to EvpC significantly, suggesting that this C-terminal ordered region is not essential for EvpC interaction. Further deletion up to residue Pro143 (EvpP_1–142_) caused a significant reduction in the interaction between EvpP and EvpC (Figure 6). This result suggests that the C-terminal disordered region between residues Pro143 and Ile168 is essential for the interaction between EvpP and EvpC. The ordered region containing both the 10-kDa and 4-kDa protease resistant fragments, as well as the disordered region between them, is not sufficient for the interaction between EvpP and EvpC.

## Discussion

### EvpP is a potential T6SS effector with substantial disorder

Many proteins are intrinsically disordered or equipped with disordered regions so that they can decouple specificity from affinity and achieve faster rates of association and dissociation [38], [39]. The disordered nature of the protein also allows multiple protein targets through the induced-fit model [40] and the disordered region usually undergoes folding or becomes ordered upon interaction [41]. In the case of T3SS effectors, a partially disordered conformation is necessary to enable passage through the narrow channel of the secretion needle [42]. A specific chaperone is required for each T3SS effector protein to keep it in a non-native or unfolded conformation ready for secretion. For instance, the disordered chaperone binding region of YopE becomes ordered upon binding to the SycE chaperone [43]. YopE is kept in an extended and non-native conformation which wraps around the chaperone and readies the protein for secretion through the narrow channel of the YscF needle [44].

In *E. tarda*, the T6SS gene cluster harbors a secreted protein EvpP, which could be a potential effector protein essential for bacterial virulence. As most T3SS effectors contain a substantial proportion of disordered regions in the absence of the chaperone, we sought to determine whether this is also the case for EvpP and, if in existence, whether these disordered regions are involved in its interaction with EvpC, the only known interaction partner of EvpP. Indeed, a significant proportion of EvpP is disordered (∼40%). A specific disordered region at the C-terminal region of EvpP, spanning from residues Pro143 to Ile168, was found to be involved in its interaction with EvpC, which also suggests that EvpP could be a potential classic type of T6SS effector that requires interaction with EvpC for secretion [24]. EvpC may also act as a chaperone to stabilize EvpP through these interactions.

Up to date, most identified T6SS effectors are anti-bacterial effectors targeting cell wall of neighboring bacterial cells. In *Pseudomonas aeruginosa*, peptidoglycan degrading effectors, Tse1-3 (type VI secretion exported 1–3) toxins are known to kill a broad range of bacteria through secretion by the T6SS [45]. Four families of cell wall-targeting peptidoglycan amidase enzymes, Tae1-4 (type VI amidase effector), have also been identified as substrates of the T6SS-1 in *Burkholderia thailandensis* using mass spectrometry [46]. These anti-bacterial effectors are relatively ordered as many of them can be crystallized for structure determination [47]–[50]. They carry out their function by enzymatically digesting bacterial peptidoglycan without the need to interact with proteins of the host bacteria. In contrast, no anti-bacterial T6SS effectors were identified in *E. tarda* (unpublished results) and no homologues of Tse1 (*Pseudomonas*), Tse3 (*Psuedomonas*), Tae3 (*Ralstonia*) and Tae4 (*Salmonella*) were found in *E. tarda* as confirmed by BLAST search. EvpP is likely a potential effector against eukaryotic host with no known enzymatic activity. The substantial amount of disordered regions may allow EvpP to interact easily with host targets to manifest virulence. To confirm that EvpP is a *bone fide* T6SS effector, further experiments, such as TEM-1 β-lactamase translocation assay or western blots using anti-EvpP antibody, are needed to verify that EvpP is transferred into the target host cells.

### Differences between T3SS and T6SS effector in chaperone requirement and degree of folding

Unlike many T3SS effectors, the currently identified classic type T6SS effectors, for example, Tse1-3, do not appear to require a dedicated chaperone to keep them soluble and in an unfolded conformation. Their interaction with the HcpI, however, is still required for secretion [22]. During the course of our study, we also noticed that there are significant differences in the degree of unfolding between EvpP and known T3SS-secreted substrates. The ^1^H-^15^N HSQC spectrum of EvpP showed that the ordered region of EvpP contains residues with NH chemical shifts, widely ranging from 7.0 to 10.0 ppm, which suggests a folded conformation with varying molecular environments. In contrast, the ^1^H-^15^N HSQC of a classic T3SS substrate, ExsE, showed a typical unfolded conformation with peaks ranging narrowly from 7.8 to 8.6 ppm in the absence or presence of the chaperone, ExsC [51]. The crystal structure of the ExsC-ExsE complex showed that ExsE wrapped around the dimeric ExsC chaperone in a way similar to that of the SycE-YopE complex [52].

One probable explanation for this difference could be the variation in the internal diameter of the HcpI ring and that of the T3SS secretion needle. Six units of HcpI form a ring with an internal diameter of ∼40 Å [53]. This diameter is twice that of the T3SS needle tunnel, which has an internal diameter of ∼20 Å. The T3SS needle also contains a narrower channel section, with an internal diameter of only 10 Å [42]. The wider diameter of the HcpI ring allows passage of substrates of a larger molecular weight and more folded conformation than does the T3SS. The T3SS substrate, ExsE, on the other hand is smaller (8.7 kDa) and is in a highly unfolded conformation in the absence or presence of the chaperone ExsC [51]. Thus, given the differences in chaperone requirement and the degree of effector folding, it is likely T3SS and T6SS use different mechanisms for substrate transport.

### An effector or a regulator?

EvpP is essential for virulence of *E. tarda* PPD130/91 and the *evpP* ORF is regulated by a dedicated promoter. Interestingly, EvpP lacks homology with many of the currently identified, peptidoglycan peptidase T6SS effectors. Despite this, the disordered nature of EvpP as well as its interaction with EvpC strongly suggests that EvpP is a secreted substrate of T6SS. These findings, however, cannot prove whether EvpP is an effector or a regulator. The intrinsically disordered substrate, ExsE, secreted from the T3SS of *P. aeruginosa*, forms a tight complex with the ExsC chaperone under non-inducing conditions [51], whereas the transcription factor ExsA forms a complex with the anti-activator ExsD. Under inducing conditions, ExsE is secreted by the T3SS; this causes a reduction in intracellular ExsE levels, which in turn favors the formation of the ExsD-ExsC complex and dissociation of the ExsD-ExsA complex, thus releasing ExsA for binding to promoter to up-regulate T3SS expression [51]. In this case, T3SS-secreted ExsE acts more like a regulator than an effector. Future experiments, such as translocation assays, will be required to confirm that EvpP is indeed transferred into the host cells. The structure of the complex formed between EvpC and EvpP would provide insight into the mechanism of EvpP transport and aid in the design of small molecule inhibitors that could disrupt the secretion of EvpP from T6SS. This therapeutic approach could be applied not only against *E. tarda* but also other pathogenic bacteria to prevent bacterial infections and diseases.

## Supporting Information

File S1 **Figure S1.** Raw data for dynamic light scattering experiment on EvpP. The polydispersity index is ranged from 0.0 to 0.1. **Figure S2.** SDS PAGE showing cross-linking of EvpP with glutaraldehyde at room temperature for differnet time periods. **Figure S3.** Sequence alignment of EvpP protein from different bacterial strains. **Figure S4.** Urea denaturation curves of wild type and mutant EvpP monitored by fluorescence.
(PDF)
