# Supplementary material for: A Disordered Region in the EvpP Protein from the Type VI Secretion System of Edwardsiella tarda is Essential for EvpC Binding
Source: PLoS One. 2014 Nov 17;9(11):e110810. doi: 10.1371/journal.pone.0110810 (PMC4234509; doi:10.1371/journal.pone.0110810)
Supplement: File S1 — Figure S1. Raw data for dynamic light scattering experiment on EvpP. The polydispersity index is ranged from 0.0 to 0.1. Figure S2. SDS PAGE showing cross-linking of EvpP with glutaraldehyde at room temperature for differnet time periods. Figure S3. Sequence alignment of EvpP protein from different bacterial strains. Figure S4. Urea denaturation curves of wild type and mutant EvpP monitored by fluorescence. (PDF) [file pone.0110810.s001.pdf]

Msr#	Time(s)	Temp(C)	Radius(nm)	Polyd(nm)	PolydIndx	MW(KDa)	%Mass	Baseline	Sos Error
1	10.0	20.1	2.96	0.413	0.02	42.8	100.0	1.000	6.27
2	20.0	20.1	2.92	0.195	0.00	41.2	100.0	1.000	2.20
3	30.0	20.1	3.06	0.969	0.10	46.3	100.0	1.001	4.94
4	40.0	20.0	3.00	0.258	0.01	43.9	100.0	1.000	4.60
5	50.0	20.0	2.98	0.732	0.06	43.3	100.0	1.000	4.65
6	60.0	20.0	3.05	0.772	0.06	45.6	100.0	1.001	7.50
7	70.0	20.0	2.97	0.686	0.05	42.8	100.0	1.001	3.65
8*	80.0	20.0	3.44	1.26	0.13	60.7	100.0	1.022	33.1
9	90.0	20.0	3.09	1.03	0.11	47.3	100.0	1.000	7.71
10	100.0	20.0	3.07	0.288	0.01	46.5	100.0	1.000	5.63
11	110.0	20.0	3.03	0.831	0.08	44.9	100.0	1.000	4.16
12	120.0	20.0	3.01	0.693	0.05	44.3	100.0	1.000	2.95
Aves:									
Mono		20.0	3.01	0.624	0.05	44.4	100.0	1.000	4.93
Bi-1		0.0	0.000	----	----	0.000	0.0	0.000	0.000
Bi-2			0.000			0.000	0.0		

Figure S1. Raw DLS data for EvpP. For most of the data points, the polydispersity index (PDI) is ranged from 0.0 to 0.1 with percentage polydispersity ranged from 0-31% (% polydispersity $\sqrt{PDI} \times 100\%$,, suggesting that EvpP is a mono-dispersed dimeric species with a M.W. ~44 kDa.

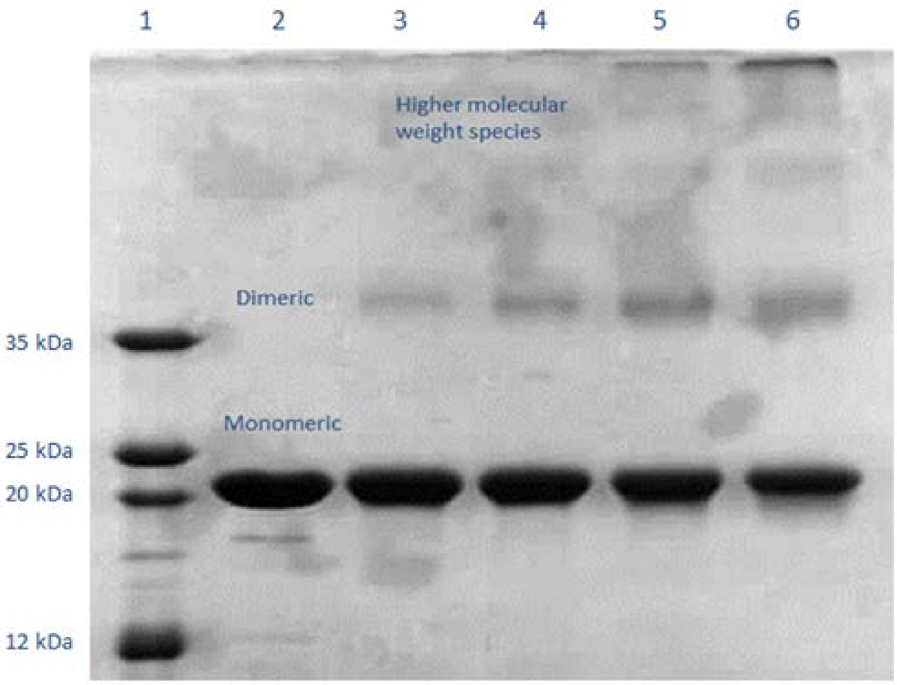

Figure S2. Cross-linking of EvpP with glutaraldehyde. Cross-linking of 50 µM EvpP with 2.3% glutaraldehyde at room temperature for time periods of (2) 0 min; (3) 15 min; (4) 30 min; (5) 45 min; and (6) 60 min. The molecular weight marker is loaded in lane (1).

```
Aeromonas     MLTLNSELEWSDVGSICTDQKAKTYVSDAFNIAYGQPTKELLPAGTALYKFNGFSSLARS 60
EIB202        MLTLNSELEWSDVGSICTDQKAKTYVSDAFNIAYGQPTKELLPAGTALYKFNGFSSLARS 60
080813        MLTLNSELEWSGVGSICTDQKAKTYVSDAFNIAYGQPTKELLPAGTALYKFNGFNSLARS 60
FL6-60        ------------------------------------------------------------
130/91        MSILNSELDWSHVGSISTG--PGTVVSDAFNISYGLPTKELLPAGTALYKFNGFSSLARP 58

Aeromonas     PITDDSPLSPWWSPVQPFRYDGGLRQRMLIAKQNGVSMREWGRLTSVIKENWSSLDFLLE 120
EIB202        PITDDSPLSPWWSPVQPFRYDGGLRQRMLIAKQNGVSMREWGRLTSVIKENWSSLDFLLE 120
080813        PITDDSPLSPWWSPVQPFRYDGGLRQRMLIAKQNGVSMREWGRLTSVIKENWSSLEYLLE 120
FL6-60        ---------------------------MLIAKQNGVSMREWGRLTSVIKENWSSLDFLLE 33
130/91        PITDDTPLSPWWSPVQPFRHDGGLQQRMLVAKLNGVSMREWGRLTSVIKENWSSLDHLLE 118

Aeromonas     IVLKIPVYAWFGGFKGMSRIDNGMTSKRNITLEQKGRSSMLPGGATQFYIPNLTVGHISS 180
EIB202        IVLKIPVYAWFGGFKGMSRIDNGMTSKRNITLEQKGRSSMLPGGATQFYIPNLTVGHISS 180
080813        ITLKVPVYAWFGGFKGMSRIDNGMTSRRNITLEQKGRSSMLPGGATQFYIPNLTVGHISS 180
FL6-60        IVLKIPVYAWFGGFKGMSRIDNGMTSKRNITLEQKGRSSMLPGGATQFYIPNLTVGHISS 93
130/91        IVLKVPVYAWFGGFKGMSRIDNGMPSKRNITLEQKGRGSNLPGGATQFYIPNLTVGHISS 178

Aeromonas     HQFSILK 187
EIB202        HQFSILK 187
080813        HQFSILK 187
FL6-60        HQFSILK 100
130/91        HNFSALK 185
```

Figure S3. Sequence alignment of EvpP protein from different bacterial strains. Sequence comparison of EvpP proteins from *Aeromonas hydrophila* (ACR24240.1), *Edwardsiella tarda* EIB202 (ACR24235.1), *Edwardsiella tarda* 080813 (ACR24238.1) and *Edwardsiella tarda* FL6-60 (ADM42303.1), and *Edwardsiella tarda* 130/91 (ABW69080.1). The numbers within parentheses are GenBank accession codes. Residues that are unique to *E. tarda* 130/91, as compared with other homologues, are highlighted in red.

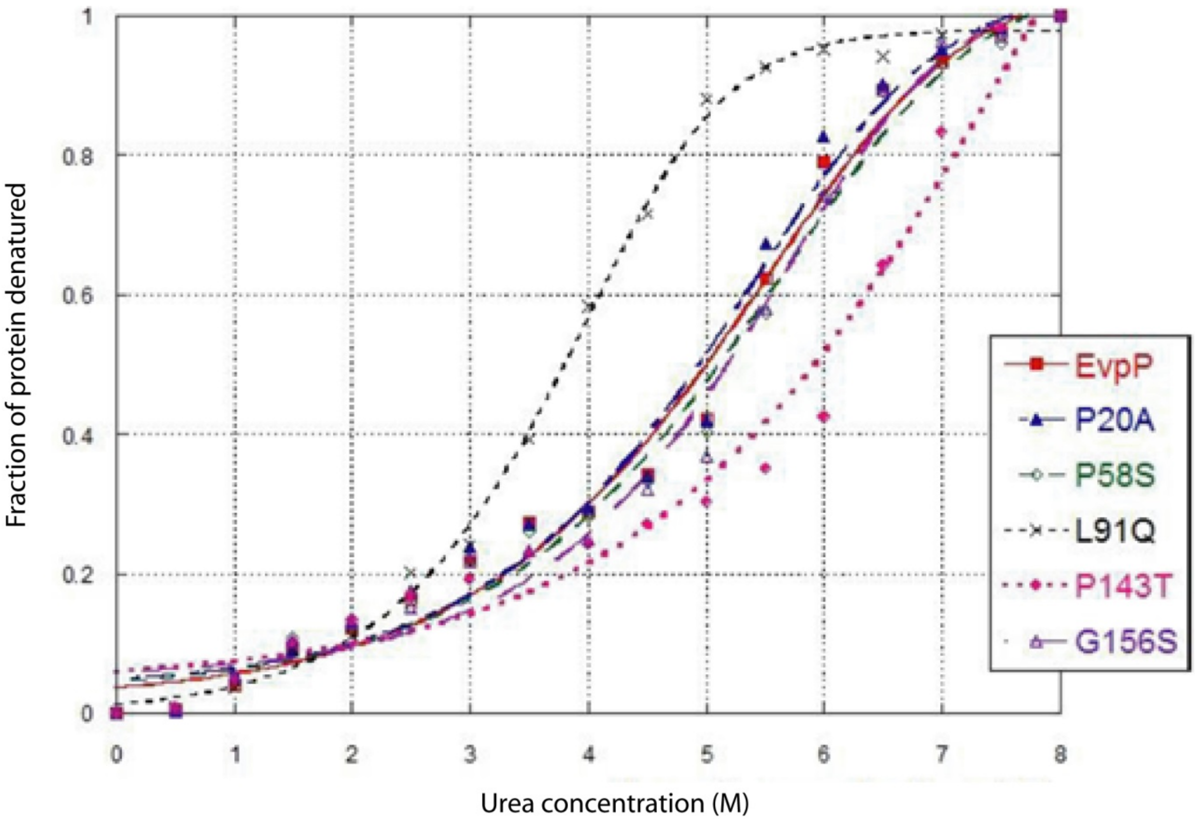

Figure S4. Urea denaturation curves of wild type and mutant EvpP. Urea denaturation is performed to determine the relative stability of wild type EvpP as compared with the P20A, P58S, L91Q, P143T and G156S EvpP mutants. Denaturation of L34Q was not performed because this particular mutant could not be expressed.
